# Binding of the Inhibitor Protein IF_1_ to Bovine F_1_-ATPase

**DOI:** 10.1016/j.jmb.2010.12.025

**Published:** 2011-02-25

**Authors:** John V. Bason, Michael J. Runswick, Ian M. Fearnley, John E. Walker

**Affiliations:** The Medical Research Council Mitochondrial Biology Unit, Hills Road, Cambridge CB0 2XY, UK

**Keywords:** GFP, green fluorescent protein, mitochondria, F_1_-ATPase, inhibitor protein, mutations, binding site

## Abstract

In the structure of bovine F_1_-ATPase inhibited with residues 1–60 of the bovine inhibitor protein IF_1_, the α-helical inhibitor interacts with five of the nine subunits of F_1_-ATPase. In order to understand the contributions of individual amino acid residues to this complex binding mode, N-terminal deletions and point mutations have been introduced, and the binding properties of each mutant inhibitor protein have been examined. The N-terminal region of IF_1_ destabilizes the interaction of the inhibitor with F_1_-ATPase and may assist in removing the inhibitor from its binding site when F_1_F_o_-ATPase is making ATP. Binding energy is provided by hydrophobic interactions between residues in the long α-helix of IF_1_ and the C-terminal domains of the β_DP_-subunit and β_TP_-subunit and a salt bridge between residue E30 in the inhibitor and residue R408 in the C-terminal domain of the β_DP_-subunit. Several conserved charged amino acids in the long α-helix of IF_1_ are also required for establishing inhibitory activity, but in the final inhibited state, they are not in contact with F_1_-ATPase and occupy aqueous cavities in F_1_-ATPase. They probably participate in the pathway from the initial interaction of the inhibitor and the enzyme to the final inhibited complex observed in the structure, in which two molecules of ATP are hydrolysed and the rotor of the enzyme turns through two 120° steps. These findings contribute to the fundamental understanding of how the inhibitor functions and to the design of new inhibitors for the systematic analysis of the catalytic cycle of the enzyme.

## Graphical Abstract

**Figure:**
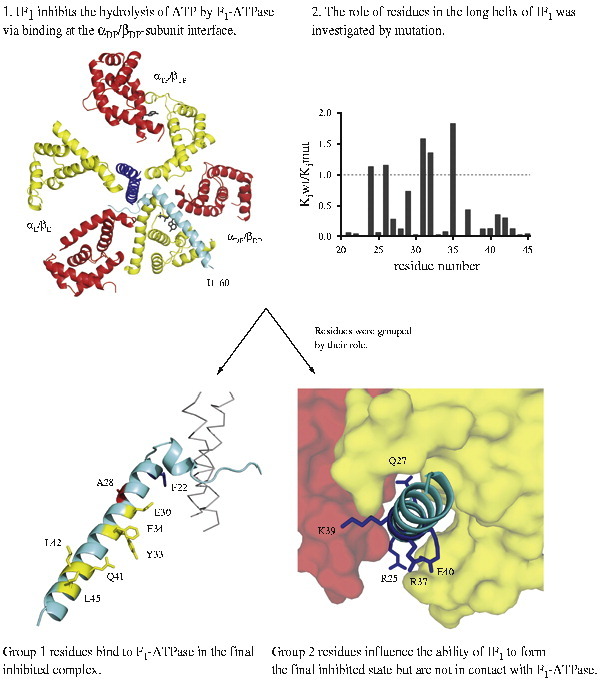

## Introduction

**Fig. 1:**
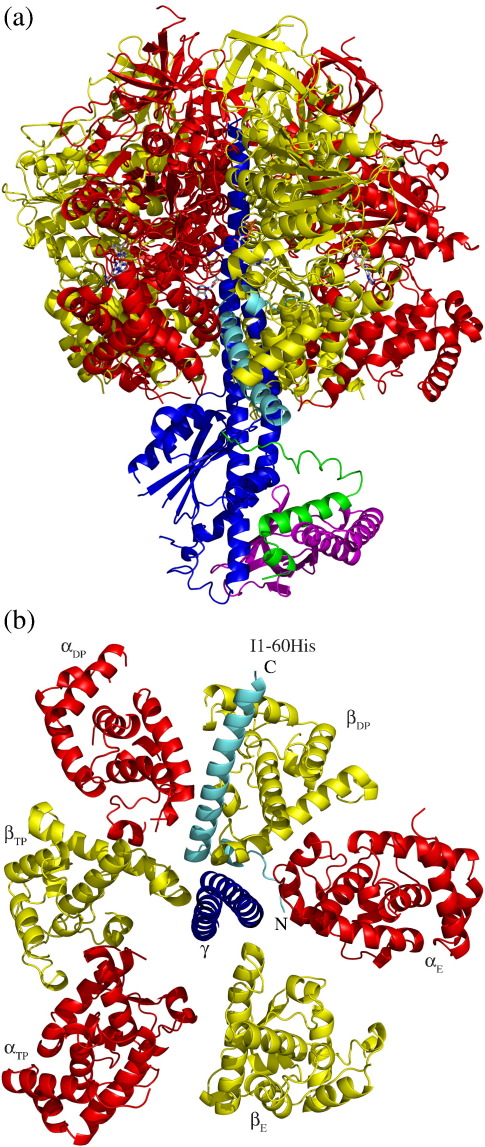
The structure of bovine F_1_-ATPase inhibited with residues 1–60 of the bovine inhibitor protein IF_1_. The α-, β-, γ-, δ-, and ɛ-subunits are shown in ribbon representation in red, yellow, dark blue, magenta, and green, respectively. The inhibitor protein is light blue. (a) Side view of the complex towards the catalytic interface between the α_DP_- and β_DP_-subunits where the inhibitor protein is bound; (b) cross-sectional view of the C-terminal domains of the α- and β-subunits looking up along the axis of the γ-subunit showing interactions of the inhibitor protein with the subunits of F_1_-ATPase.

Mitochondria contain a small basic inhibitor protein known as IF_1_, which binds to the F_1_ catalytic domain of the F_1_F_o_-ATP synthase (F-ATPase) complex. However, it has no effect on F-ATPases from eubacteria or chloroplasts. This inhibitor protein inhibits the ATP hydrolase activity of the mitochondrial enzyme, but unlike almost all other inhibitors of F_1_F_o_-ATP synthase, which prevent both hydrolysis and synthesis, it is a unidirectional inhibitor and does not inhibit ATP synthesis.^1–3^ Bovine IF_1_ is 84 amino acids long,^4^ and its structure is a homo-dimer held together by an antiparallel coiled coil of α-helices involving residues 49–81 in the C-terminal region of each monomer.^5^ The N-terminal regions, which are also largely α-helical, contain the inhibitory sector. Each inhibitory sector in the dimer binds to one of the three catalytic sites in the F_1_ domains in two separate F_1_F_o_ complexes by a mechanism in which each complex hydrolyses two molecules of ATP.^2,6^ A monomeric fragment of IF_1_ consisting of residues 1–60 (I1-60) inhibits a single F_1_F_o_-ATPase complex. In a structure of bovine F_1_-ATPase inhibited with I1-60, known as F_1_-I1-60,^6^ the inhibitor is bound at a catalytic interface between the β_DP_- and α_DP_-subunits (see Fig. 1), and ADP–magnesium complexes are trapped in the catalytic sites of both the β_DP_- and β_TP_-subunits, whereas the catalytic site in the β_E_-subunit is unoccupied except for a phosphate molecule bound to the P-loop of the subunit. In this complex, the inhibitor protein appears to be bound to F_1_-ATPase predominantly via residues 21–46 in the longer of its two α-helices. These residues occupy a deep groove in the cleft between the C-terminal regions of the β_DP_- and α_DP_-subunits, whereas residues 47–50 extend beyond the external surface of the F_1_-domain. This groove is lined with segments of α-helices and loops in the C-terminal domains of the β_DP_- and α_DP_-subunits and with other similar features in the C-terminal domains of the β_TP_- and α_E_-subunits. Residues 14–18 of IF_1_ make a second α-helix, which is linked to the longer α-helix by an extended region containing residues 19 and 20. The shorter α-helix and the region linking it to the longer α-helix make further interactions with α-helical regions in the γ-subunit in the central stalk, or rotor, of the enzyme. Residues 1–7 were not resolved in the structure, but residues 8–13 have an extended structure, which interacts with the nucleotide binding domain of the α_E_-subunit. Hence, I1-60 interacts with five of the nine subunits of F_1_-ATPase.

**Fig. 2:**
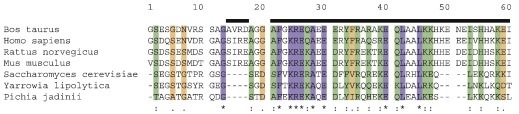
Alignment of the sequence of residues 1–60 of bovine IF_1_ with equivalent portions of F_1_-ATPase inhibitor proteins from other species. The purple, yellow, and green stripes denote strictly conserved, highly conserved, and poorly conserved residues, respectively. The black lines above the sequences mark α-helical regions in bovine IF_1_. The Expasy accession numbers for inhibitor proteins from *Bos taurus*, *Homo sapiens*, *Rattus norvegicus*, *Mus musculus*, *Saccharomyces cerevisiae*, and *Pichia jadinii* are P01097, Q90112, Q03344, Q35143, P01097, and P09940, respectively.

Many of the amino acid residues of IF_1_, especially in its longer α-helix, are conserved strictly, and others have been substituted conservatively during evolution (see Fig. 2).^7^ Therefore, as described here, in order to understand their relative contributions to the binding of IF_1_ to F_1_-ATPase, several N-terminally truncated forms of I1-60 have been made, and point mutations have been introduced into its longer α-helix. The binding properties of the mutated proteins have been studied quantitatively.

## Results and Discussion

### Expression and characterization of inhibitor proteins

The C terminus of residues 1–60 of bovine IF_1_ was fused to the green fluorescent protein (GFP) in order to enhance its expression in *Escherichia coli* with a six-histidine tag to facilitate protein purification. This protein is known as I1-60GFPHis, and most of the mutations were introduced into this version of IF_1_. Five N-terminally truncated versions of I1-60GFPHis were made, and point mutations were introduced into residues 21–45. Twelve inhibitor proteins with some of the same point mutations were made in a version of I1-60 with a six-histidine tag fused directly to its C terminus and lacking a GFP domain.

All of the inhibitor proteins were isolated by nickel affinity chromatography. Their purities were demonstrated by SDS-PAGE (Supplementary Fig. S1), and their molecular masses were characterized by electrospray ionization mass spectrometry (Supplementary Table S1). The experimentally measured mass values corresponded to the calculated values, with one exception where the C-terminal His-tag lacked one histidine residue. It is unlikely that this change had any impact on the inhibitory properties of the protein.

As described in Materials and Methods, the inhibitory properties of the various inhibitor proteins were assessed by measuring their binding and dissociation rate constants, *k*_on_ and *k*_off_, with F_1_-ATPase.^8^ A detailed description of the experimental determination of *k*_on_ and *k*_off_ values for the inhibitors containing the point mutations F22Y, E31A, and F34A is provided in Materials and Methods, with example data sets shown in Supplementary Figs. S2 and S3 and Supplementary Table S2. In one case, I1-60His F22A, the addition of the inhibitor protein to F_1_-ATPase gave rise to a linear, and not an exponential, decrease in the activity of the enzyme; consequently, the *k*_on_ and *k*_off_ values were not determined. Therefore, the value of *K*_i_ was estimated from a Lineweaver–Burke plot (Supplementary Fig. S4). The values determined for *k*_on_, *k*_off_, and *K*_i_ of the various inhibitors are summarized in Supplementary Tables S3 and S4. These values include those for the wild-type I1-60GFPHis inhibitor protein, which has a dissociation constant (*K*_i_ value) of 65 nM^− 1^, and for the inhibitor protein I1-60 lacking both the His tag and the GFP domain, where the *K*_i_ value of 30 nM^− 1^ illustrates that the GFP moiety in I1-60GFPHis weakens the ability of the inhibitor to reach the site of inhibition. Nevertheless, I1-60GFPHis is a potent inhibitor, and it provides an appropriate comparator for the various mutant derivatives.

Where the introduction of a point mutation reduced the inhibitory potency of the protein substantially, the mutated proteins were made without the GFP domain, which is dominated by β-structures, and the circular dichroism spectrum of each protein was recorded. The circular dichroism spectrum is recognised as a sensitive and accurate means of assessing the α-helical content of a predominantly α-helical protein.^9^ In Supplementary Fig. S5, the spectra for the proteins with the point mutations A21G, F22A, R25A, E30A, Y33A, F34A, R35A, Q41A, L42A, A43V, A44V, and L45A are compared with the spectrum of I1-60His (also lacking a fused GFP domain). The spectra are very similar, and there was no evidence that the introduction of any of the mutations had disrupted the long α-helix of I1-60His (Supplementary Fig. S6). Therefore, the effects of the mutations on the binding of the inhibitor protein can be attributed directly to changes associated with the mutated amino acid itself.

### Role of the N-terminal region of IF_1_

**Fig. 3:**
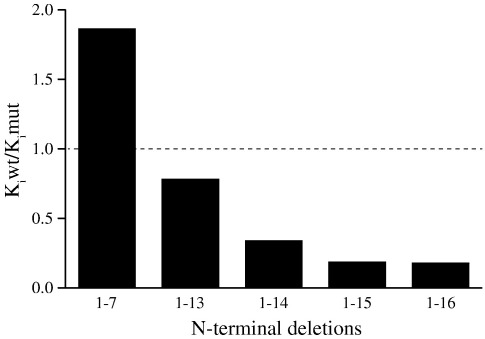
Influence of N-terminal deletions on the binding of the inhibitor protein I1-60GFPHis to bovine F_1_-ATPase. The data are taken from Supplementary Table S4. For an explanation of the quotient *K*_i_mut:*K*_i_wt, see the text.

In the structure of F_1_-I1-60, residues 1–7 were not resolved, residues 8–13 have an extended structure, and residues 14–18 form a short α-helix linked by residues 19 and 20 to the longer α-helix, which starts at residue 21.^6^ In the work presented here, five N-terminally truncated inhibitor proteins were made lacking residues 1–7, 1–13, 1–14, 1–15, and 1–16. All of these truncated inhibitors had modestly higher *k*_on_ values in comparison with wild-type I1-60His, whereas the value of *k*_off_ did not increase substantially until at least 14 residues had been deleted (Supplementary Table S3). The effects of these mutations are summarized in Fig. 3 as the quotient of the dissociation constants of the wild-type and mutant proteins, *K*_i_wt:*K*_i_mut. Values of the quotient that are greater and less than unity correspond to mutant proteins with increased and decreased binding to F_1_-ATPase, respectively. The values that were calculated (see also Supplementary Table S3) demonstrate that residues 1–7 of I1-60His do not contribute to the binding of IF_1_ to F_1_-ATPase, and their deletion increased the binding energy of I1-60His (Supplementary Table S3), probably because the confinement of the N-terminal region of the protein in the aqueous cavity in the core F_1_-ATPase decreases its entropy. The inhibitor protein I1-60GFPHis Δ1-7 is the most potent inhibitor of all those that were made, with the exception of I1-60GFPHis with the point mutation Y33W (Supplementary Table S4). The role of residues 1–7 of I1-60His may be to destabilize the interaction between the inhibitor protein and F_1_-ATPase, allowing it to leave its inhibitory site when F_1_F_o_-ATPase switches from the inhibited state to the active ATP synthesising state. The roles of residues 8–12 were not investigated, but deletion of residues beyond residue 12 weakened the interaction of the inhibitor with F_1_-ATPase progressively up to residue 15, where *K*_i_wt:*K*_i_mut reached a local minimum. The role of residues 13–20 seems to be to help to impede the dissociation of the inhibitor protein by interacting with the coiled-coil α-helical region of the γ-subunit.

### Effects of point mutations in the long α-helix of I1-60

**Fig. 4:**
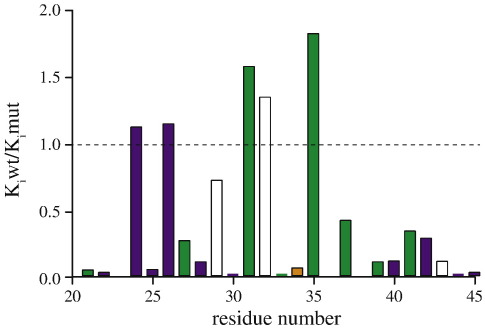
Influence of point mutations in the long α-helix of the inhibitor protein I1-60GFPHis on its binding to bovine F_1_-ATPase. The mutations and their quantitative effects on binding are given in Supplementary Table S4. *K*_i_wt and *K*_i_mut are the values for wild-type and mutant proteins, respectively. Strictly conserved, highly conserved, poorly conserved, and unconserved residues are shown in purple, yellow, green, and white, respectively. The unconserved residues G23, A36, and A38 were not mutated.

With the exceptions of the unconserved residues G23, A36, and A38, the roles of the amino acids in the long α-helix of I1-60 between residues 21 and 45 were investigated by changing non-alanine residues to alanine and by changing alanine residues to glycine (residue 21) or valine (residues 28, 43, and 44). Additionally, aromatic amino acids (F22, Y33, and F34) were substituted conservatively. The effects of many of these mutations are summarized in Fig. 4 as *K*_i_wt:*K*_i_mut values (see also Supplementary Table S4). They fall into three broad categories, namely, those inhibitor proteins where the mutation had a strong effect (*K*_i_wt:*K*_i_mut = 0–0.12), those where the mutation had an intermediate effect (*K*_i_wt:*K*_i_mut = 0.25–0.7), and those where the mutation had either little or no effect or an apparent stimulatory effect on the inhibitory properties of the protein (*K*_i_wt:*K*_i_mut > 1).

#### Residues where mutation affects binding strongly (*K*_i_wt:*K*_i_mut = 0–0.12)

**Fig. 5:**
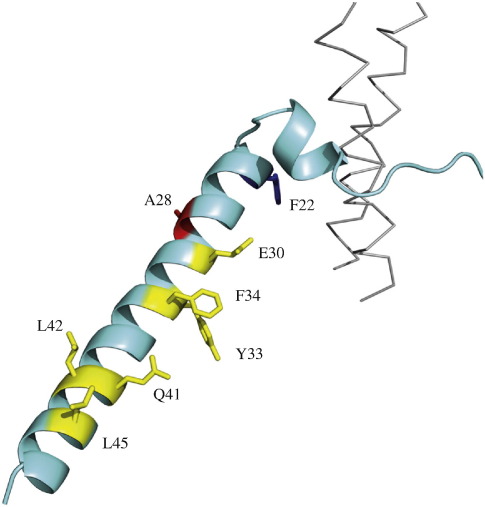
Residues in the long α-helix of the bovine inhibitor protein that contribute to its binding by interacting directly with subunits of bovine F_1_-ATPase. The inhibitor protein from residues 8–50 is pale blue, and α-helical regions (residues 14–18 and 21–50) are shown in ribbon representation. The extended N-terminal region (residues 8–13) and the shorter α-helix snake around two antiparallel α-helical regions (residues 1–23 and 232–251) in the γ-subunit, which is part of the central stalk or rotor of the enzyme. Residues with yellow side chains interact with the β_DP_-subunit; the residue with a red side chain interacts with the α_DP_-subunit; and the residue with a blue side chain interacts with the β_TP_-subunit.

Ten of the thirteen mutated residues in this category are either strictly conserved (F22, R25, A28, E30, F34, E40, and L45) or highly conserved (A21, Y33, K39); one other residue, F34, is poorly conserved; and A43 and A44 are unconserved. Most of the residues in the category fall into two distinct groups, 1 and 2. Group 1 consists of those residues that contribute to the binding energy of IF_1_ in the final inhibited complex seen in the F_1_-I1-60His structure, namely, residues F22, A28, E30, Y33, F34, and L45 (Fig. 5). The roles of F22, Y33, and F34 were studied by substituting them with larger aromatic residues. The mutations F22Y, F22W, and F34Y decreased the affinity of the inhibitor for F_1_-ATPase, whereas Y33W increased it (Supplementary Table S4). Group 2 contains those residues (A21, R25, K39, E40, A43, and A44) that, although they do not evidently contribute to binding in the structure of the final inhibited complex since they are in aqueous space, nonetheless influence strongly the ability of IF_1_ to form the final inhibited state.

#### Residues where mutation has an intermediate effect (*K*_i_wt:*K*_i_mut = 0.25–0.7)

Of the four mutated residues where there was an intermediate effect on *K*_i_wt:*K*_i_mut (Q27, R37, Q41, and L42), L42 is strictly conserved and Q27, R37, and Q41 are highly conserved. L42 and Q41 belong to group 1 and Q27 and R37 belong to group 2.

#### Residues where mutation had either little or no effect or a stimulatory effect (*K*_i_wt:*K*_i_mut > 1)

Of the six residues in this category (K24, E26, E29, E31, R32, and R35), two are strictly conserved (K24 and E26) and two others (E31 and R35) are highly conserved. These four residues form a third distinctly separate group of residues, group 3, which are strictly or highly conserved, but their mutation has no effect on forming either the final inhibited state (group 1 residues) or intermediate state in the pathway leading to the formation of the final inhibited state (group 2 residues). The fifth and sixth members of the category, E29 and R32, are unconserved. They also seem to have no important role in the inhibition process, but their lack of conservation argues that whatever their role may be, they are less important than the four members of group 3.

#### Roles of group 1 residues

The residues belonging to this group are F22, A28, E30, Y33, F34, L42, L45, and Q41 (Fig. 5). With the exception of F22 and A28, which sit in hydrophobic pockets formed by residues I390 and L391 from helix 1 in the β_TP_-subunit and residues A402 and F403 from helix 2 in the α_DP_-subunit, respectively, they make interactions, many of them hydrophobic, with helices 2 and 6 in the C-terminal domain of the β_DP_-subunit (see Fig. 5).^6^ Residues Y33, L42, and L45 are in contact with hydrophobic surfaces in helices 2 and 6, and F34 occupies a hydrophobic pocket made from residues V404, S405, and R408 of the β_DP_-subunit.^6^ As expected, mutation of the non-alanine residues to alanine decreased the affinity of the inhibitor for F_1_-ATPase, presumably by decreasing the area of hydrophobic contact. In addition, the introduction of bulkier aromatic side chains via the mutations F22W, F22Y, and F34Y decreased the affinity of the inhibitor, presumably because the bulkier side chains of tyrosine and tryptophan cannot be accommodated in the hydrophobic binding pockets. In contrast, the substitution of Y33 by tryptophan increased the binding of the inhibitor, presumably by increasing the area of hydrophobic contact with the β_DP_-subunit. The only charged residue in the long α-helix to contribute in a major way to the binding of the inhibitor protein to F_1_-ATPase is residue E30, which forms a salt bridge with R408 in helix 2 in the C-terminal domain of the β_DP_-subunit.^6^ In addition, the polar residue Q41 contributes to binding by interaction with D450 in the loop linking helices 5 and 6 in the C-terminal domain of the β_DP_-subunit. In the mutant inhibitor containing the mutation A28V, the bulkier valine residue will clash with helix 2 in the α_DP_-subunit in F_1_-ATPase, thereby decreasing the affinity of the inhibitor.

#### Roles of group 2 residues

**Fig. 6:**
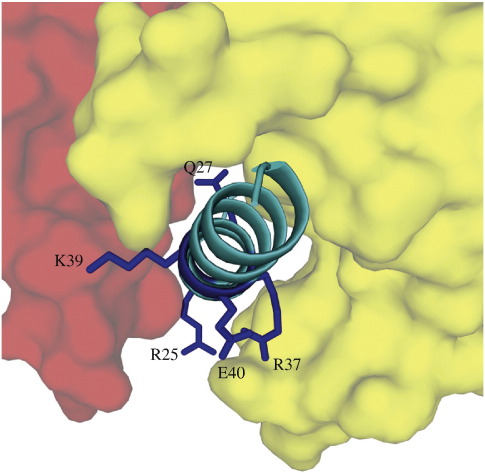
Charged residues in the long α-helix of the bovine inhibitor protein that are required for the binding of the inhibitor to F_1_-ATPase but do not interact with F_1_-ATPase in the structure of the inhibited complex. The long α-helix of IF_1_ (residues 21–50; light blue) occupies an aqueous cleft between the C-terminal domains of the β_DP_- and α_DP_-subunits (yellow and red, respectively). The side chains of amino acids that do not interact with the β_DP_- and α_DP_-subunits, but nonetheless are required for the formation of the inhibited complex, are dark blue.

The residues in this group are A21, R25, Q27, R37, K39, E40, A43, and A44. In the crystal structure of F_1_-I1-60His, the charged residues of this group do not interact directly with F_1_-ATPase, and each of them occupies an aqueous cavity (see Fig. 6).^6^ Nonetheless, the mutation of each of these residues affected the binding of I1-60GFP to F_1_-ATPase, indicating that they play roles at some as yet undefined point in the pathway of the binding of the inhibitor to the enzyme that leads to the inhibited state observed in the crystal structure (and possibly in its reversal). Neither A43 nor A44 interacts with F_1_-ATPase in the structure of bovine F_1_-I1-60His. The substitutions A43V and A44V both decreased the affinity of I1-60GFPHis for F_1_-ATPase substantially, probably because the bulkier valine residues clash with regions of F_1_-ATPase. It is possible that A43 and A44 participate in making an intermediate in the pathway leading to the final inhibited state. This conclusion is rather tentative, as is the inclusion of these two residues in group 2.

#### Group 3 residues

The residues in this group are K24, E26, E31, and R35. In the structure of bovine F_1_-I1-60His, R35 appears to form a salt bridge with E399 in the α_DP_-subunit.^6^ Therefore, it was surprising to find that the mutation R35A had little effect on binding, and thus it must be concluded that the salt bridge observed in the structure is not a significant feature. In the structure of a dimer of dimers of IF_1_, E26 forms a salt bridge with H49 in a second dimer, apparently helping to stabilize the inactive tetramer.^4^ It has been suggested that this residue may be part of a pH-sensitive switch that regulates the activity of IF_1_ by influencing its oligomeric state.^5,10,11^ The role of K24 remains obscure. In addition, it is not clear why the mutation E31A stimulated the inhibitory activity of I1-60GFPHis.

### Comparison of the inhibition of bovine and yeast F_1_-ATPases

The effects of the introduction of point mutations into IF_1_ from *Saccharomyces cerevisiae* upon their inhibitory activity with yeast F_1_-ATPase have been studied independently by measuring the effect of mutations on the inhibition of ATPase activity.^12^ It was concluded that residues F17 (equivalent to bovine F22), R20 (bovine R25), R22 (bovine Q27), E25 (bovine E30), and F28 (bovine Y33) in the yeast IF_1_ are essential for the activity of the inhibitor. In addition, residues A23, R30, R32, Q36, L37, L40, and L44 also play a role (the equivalent bovine residues are A28, R35, R37, Q41, L42, L45, and H49, respectively). With the exception of yeast R30 (bovine R35), these results are in broad agreement with the results presented here for the inhibition of bovine F_1_-ATPase by bovine IF_1_. R35 does not seem to have a role in the inhibitory activity of bovine IF_1_. Therefore, as presented elsewhere, the effects of the mutation of R30 in yeast IF_1_ have been reassessed with yeast F_1_-ATPase by measuring the *k*_on_ and *k*_off_ of the wild-type yeast inhibitor and a form carrying the mutation R30A (G. C. Robinson, J. V. Bason, M. G. Montgomery, D. Mueller, A. G. W. Leslie, and J. E. Walker, unpublished results). These measurements show that the wild-type and mutant proteins have almost identical *K*_i_ values. Residue L44 in yeast IF_1_ was also proposed to play a role in the inhibition of the yeast enzyme. The equivalent residue in the bovine inhibitor, H49, was not studied in the work described here. In the structure of bovine F_1_-I1-60 it is found in the α-helical region of I1-60 that lies beyond the external boundary of F_1_-ATPase, and in a recently determined structure of yeast F_1_-ATPase inhibited with yeast IF_1_, L44 is in a similar location (G. C. Robinson, J. V. Bason, M. G. Montgomery, D. Mueller, A. G. W. Leslie, and J. E. Walker, unpublished results). The available structural information does not suggest that this residue plays a direct role in forming the inhibited complex.

### The mechanism of binding of IF_1_ to F_1_-ATPase

**Fig. 7:**
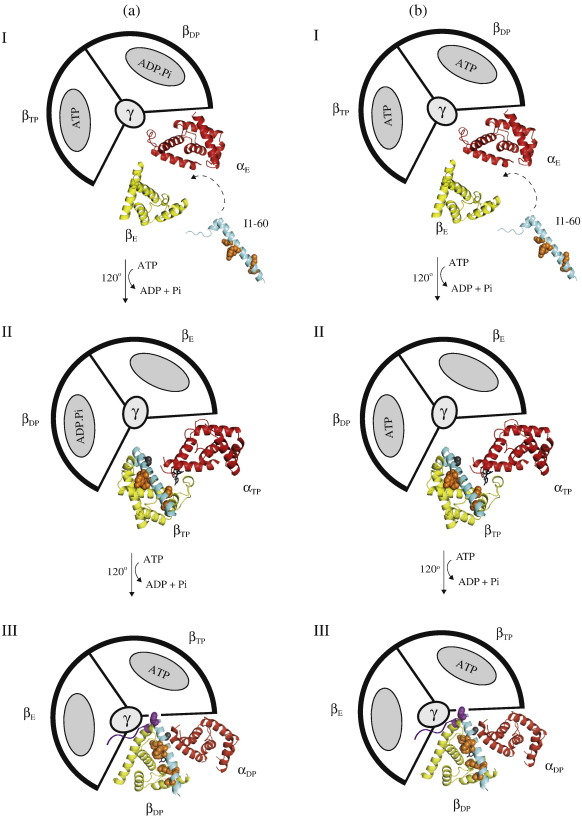
Possible schemes of the inhibition of F_1_-ATPase by the inhibitor protein I1-60. F_1_-ATPase is depicted as viewed away from the inner mitochondrial membrane from the foot of the central stalk. For simplicity, only those α- and β-subunits that form the catalytic interface, where I1-60 binds, are shown in ribbon representation. The α- and β-subunits are in red and yellow, respectively, and I1-60 is in light blue. The remaining α- and β-subunits and the γ-subunit are represented schematically, and the corresponding α-subunits have been omitted for simplicity. Panel A: In state I, IF_1_ binds first via the longer α-helix to the C-terminal domain of the β_E_-subunit in the catalytic interface between the α_E_- and β_E_-subunits when the enzyme is in the ground state [PDB code 2JDI].^13^ These initial interactions between inhibitor and enzyme involve many of the group 1 residues, E30, Y33, F34, Q41, L42, and L45 (orange), and the formation of this and subsequent intermediates may also include the group 2 charged residues, R25, Q27, R37, K39, and Q41. In state II, rotation of the γ-subunit through 120° closes the α_E_β_E_ interface, converting it to the α_TP_β_TP_ interface [PDB was made by docking I1-60 into the α_TP_-β_TP_ interface of the azide free ground state structure (PDB code 2JDI)^13^ at the same angle to which it binds to the α_DP_β_DP_ interface in the final inhibited state (PDB code 2v7q)^6^]. This α_TP_ β_TP_ interface is similar in structure but not identical to the α_DP_ β_DP_ interface, and many of the interactions observed in the structure of the final inhibited state probably will be present at this stage. They include the interactions formed by the group 1 residues of IF_1_ (E30, Y33, F34, Q41, L42, and L45; orange). Also, it is possible that the group 1 residue A28 (grey) may bind to the α_TP_-subunit at this point. However, residue F22 cannot interact with F_1_-ATPase at this stage. In state III, further rotation of the γ-subunit through 120° leads to the formation of the final inhibited structure [PDB code 2v7q],^6^ either, as depicted in scheme A, a dead end state, where an ATP molecule has been hydrolysed at the same catalytic interface that I1-60 binds, or, as shown in scheme B, a pre-hydrolysis state, where an ATP molecule has not been hydrolysed.^6^ Residue F22 (purple) can now interact with the β_TP_-subunit, completing the binding interactions between the long α-helix of IF_1_ and F_1_-ATPase. In this final inhibited state, the N-terminal region (purple) will also interact with the γ- and α_E_-subunits.

In order for IF_1_ to gain access to and bind to its complex binding site, it is reasonable to propose that the initial interaction between the inhibitor and the enzyme is formed with the C-terminal region of a β-subunit in its most accessible and open conformation during the catalytic cycle of ATP hydrolysis, namely, the one represented by the β_E_-subunit, where no nucleotide is bound (see Fig. 7). (An alternative view that the initial interaction takes place with the β_TP_-subunit has been advanced also.^13^) This initial interaction with the β_E_-subunit may involve some or all of the group 1 residues, E30, Y33, F34, Q41, L42, and L45. It is also possible that group 2 residues such as R25, Q27, R37, K39, and E40, which are not making contacts with F_1_-ATPase in the final inhibited complex, but where nonetheless mutation influences the binding of the inhibitor, are also involved in these early and intermediate binding steps. Thus far, none of them has been characterized structurally or kinetically, but it may be possible to do so by forming a “ground state” analogue complex of F_1_-ATPase with a non-hydrolysable form of ATP or with ADP and beryllium chloride^14,15^ and by estimating *k*_on_ and *k*_off_ with an inhibitor protein bound appropriately to a surface plasmon resonance chip. In order to proceed to the final observed inhibited state, the γ-subunit must rotate through 240° in two consecutive 120° steps. In the first 120° rotational step, which is driven by ATP binding at the catalytic site in the β_E_-subunit, the β_E_-α_E_ interface is converted to the β_TP_-α_TP_ interface. At this stage, most of the important interactions between the longer α-helix of IF_1_ and F_1_-ATPase, as observed in the structure of F_1_-I1-60, are likely to have formed already. Only during the second 120° rotational step driven by ATP binding to the newly generated β_E_-subunit can residue F22 of the inhibitor enter its binding site in the hydrophobic pocket in the adjacent β-subunit (the one that becomes the β_TP_-subunit in the final inhibited state) and augment the binding of the inhibitor to F_1_-ATPase (see Fig. 7). The importance of this residue in achieving the final inhibited state is reflected by the impact on binding of the introduction of the mutation F22A into the inhibitor (Supplementary Table S4). During or after this second 120° rotational step, it is likely that the interactions between the γ-subunit and the shorter α-helix (residues 14–18 of IF_1_) and the region linking it to the longer α-helix (residues 19–21) will also form.

The mechanism of the reversal of the inhibition of F_1_F_o_-ATPase by IF_1_, in response to the imposition of a proton-motive force to drive ATP synthesis, is not known. However, the restoration of ATP synthesis requires the reversal of the direction of rotation of the rotor of the enzyme from the counterclockwise direction that accompanies ATP hydrolysis, as viewed from the membrane domain of the enzyme, to the clockwise direction required for ATP synthesis. This reversal of the direction of rotation of the rotor evidently destabilizes the interactions of IF_1_ with the F_1_-domain of the inhibited enzyme leading to its eventual ejection from the inhibited catalytic interface. It seems likely that the interactions between residues 14–20 of IF_1_ with the rotor itself will be destabilized in the early part of this reversal of inhibition, and that residues 1–7 will contribute to the destabilization of the binding of IF_1_. As the inhibited catalytic interface opens in response to the reversed rotation of the rotor, the interaction of residue F22 with the β_TP_-subunit will be broken, followed at a later stage by the disruption of interactions between the long α-helix of IF_1_ and the C-terminal domain of the β-subunit that formerly had been the β_DP_-subunit in the final inhibited state, and the release of IF_1_.

### Practical implications

Knowledge about which residues of IF_1_ are most important for forming the inhibited complex has practical significance. First, because of the complexity of the mode of binding of IF_1_ to F_1_-ATPase and its high affinity for F_1_-ATPase, bovine IF_1_ is highly specific and selective for the ATPase activity of bovine F_1_F_o_-ATP synthase. These properties have been put to use in a simple affinity chromatography method for purifying the inhibited bovine complex (M. J. Runswick, J. V. Bason, I. M. Fearnley, and J. E. Walker, unpublished results). Since F_1_F_o_-ATPases are highly conserved in vertebrates, this method has been used to purify of the human, sheep, pig, rabbit, and mouse enzymes, and a similar procedure has been developed for purifying the enzyme from fungal species (T. Charlesworth, M. J. Runswick, J. V. Bason, I. M Fearnley, S. Ferguson, and J. E. Walker, unpublished results). In developing these procedures, knowledge of how to strengthen or weaken the interaction of I1-60 with the F_1_-domain by introducing appropriate mutations has proved to be very valuable. Second, a comparison of the structures of bovine and yeast F_1_-ATPases inhibited by their cognate inhibitor proteins has shown that although the inhibited complexes are very similar in many respects, there are differences as well. The most notable is that although the inhibitors bind in a similar way to F_1_-ATPases, their modes of binding are not identical; the yeast inhibitor binds at a slightly steeper angle relative to the central rotor of the enzyme than the bovine inhibitor. As a consequence of these subtle differences in binding mode, the catalytic cycle of the yeast enzyme has been arrested at an earlier stage than in the bovine inhibited complex, revealing a new intermediate state in the catalytic pathway of the enzyme (G. C. Robinson, J. V. Bason, M. G. Montgomery, D. Mueller, A. G. W. Leslie, and J. E. Walker, unpublished results). In this intermediate state, an ATP molecule has been hydrolysed, and both phosphate and magnesium have been released from the α_E_β_E_ catalytic interface, whereas the nucleotide, ADP, remains bound. This finding offers the prospect that it may be possible, using the information given in the present study, to engineer new forms of I1-60 that arrest the catalytic cycle of bovine F_1_-ATPase at other as yet uncharacterized intermediate stages and to characterize the intermediates by structural analysis.

## Materials and Methods

### Overexpression and purification of recombinant proteins

An expression plasmid encoding residues 1–60 of bovine IF_1_ with the enhanced green fluorescent protein fused to its C terminus, followed by a hexahistidine sequence, was cloned into plasmid pRun. The encoded protein is referred to as I1-60GFPHis. Single, double, and triple point amino acid mutations were introduced into this sequence with a series of pairs of synthetic complementary oligonucleotide primers containing the mutated codons and 24 bases 5′ and 3′ of the codon, respectively. This region was amplified by PCR and extended in a second PCR with primers flanking the 5′ and 3′ ends of the coding sequence for I1-60GFPHis. The template plasmid contained restriction digest sites for NdeI and HindIII 5′ and 3′ of the gene, respectively, and thus the modified sequences were restricted and religated into the predigested plasmid, pRun, to produce the requisite expression plasmids. The procedure was repeated to produce expression plasmids for mutant inhibitor proteins not containing GFP.

Cells of *E. coli* C41 (DE3)^16^ were transformed with plasmids encoding wild-type and mutant forms of I1-60GFPHis and I1-60His. Cells were grown in 2xTY medium at 37 °C. When the cell density had reached an absorbance of 0.6 at 600 nm, protein expression was induced with isopropyl-β-d-thiogalactopyranoside (0.286 mg/ml final concentration). After 18-h growth at 25 °C, cells were harvested by centrifugation (6500***g***, 10 min). They were resuspended in buffer A [20 mM Tris–HCl (pH 7.4), 10% (v/v) glycerol, 25 mM imidazole, and 0.1 M sodium chloride] and broken with a Constant Cell disruptor (Constant Cell, Daventry, UK). All subsequent steps were performed at 4 °C. Cell debris was removed by centrifugation at 265,000***g*** for 1 h. The supernatant was filtered through a Minisart membrane (pore size, 0.2 μm; Sartorius, Goettingen, Germany) and applied to a Hi-Trap nickel Sepharose column (GE Healthcare, Buckinghamshire, UK) equilibrated in buffer A. I1-60GFPHis, I1-60His, and mutant forms were eluted with a linear gradient of imidazole from 25 to 300 mM in a total volume of 100 ml of buffer A. Fractions containing the proteins were pooled and dialysed for 4 h against 2 l of buffer consisting of 20 mM Tris–HCl (pH 7.4) and concentrated to 24 mg/ml with a VivaSpin concentrator (molecular weight cutoff, 5 kDa; Sartorius, Göttingen, Germany).

### Protein analysis

The purification of proteins was monitored by SDS-PAGE in 12–22% acrylamide gradient gels. Proteins were detected by staining with Coomassie brilliant blue dye. The sequences of recombinant inhibitor proteins were verified by measurement of their molecular masses by electrospray mass spectrometry in either a triple quadrupole-time of flight mass spectrometer (Q-Tof1, Micromass-Waters, Altrincham, UK) or a Quatro Ultima triple quadrupole instrument (Micromass-Waters). Samples of the various inhibitor proteins (1–2 μM in 1–2% formic acid in 50% aqueous acetonitrile) were introduced into an electrospray interface by flow injection. Spectra were recorded from 700 to 2000 *m*/*z* (mass-to-charge ratio) and processed with Mass Lynx software (Micromass-Waters). Any discrepancies were investigated by sequencing the expression plasmid.

### Estimation of the α-helical contents of proteins

Mutant inhibitor proteins lacking the fused GFP domain were dialysed for 18 h against ultra-pure water. They were diluted to a concentration of 0.125 mg/ml and their circular dichroism spectra were recorded from 190 to 260 nm with the aid of a Jasco J-810 spectropolarimeter (Jasco Inc., Easton, MD, USA). The secondary structure of the protein was calculated with the program K2D†.

### Assay of inhibition of F_1_-ATPase

Bovine F_1_-ATPase was purified from heart mitochondria as described previously.^17^ Its ATP hydrolase activity in the presence of the various inhibitors was measured with an ATP-generating system by the addition of 2.5 μg of F_1_-ATPase (specific activity; 101 μmol/mg/min) to 1 ml of assay mixture at 37 °C. The assay mixture consisted of 50 mM potassium chloride, 50 mM Tris–HCl (pH 8.0), 2 mM magnesium chloride, 0.2 mM NADH, 1 mM phosphoenol-pyruvate, 2 mM ATP, pyruvate kinase (80 μg/ml), and lactic acid dehydrogenase (80 μg/ml).^18^ Each mutant inhibitor protein, dissolved in 20 mM Tris–HCl (pH 7.4; concentration, 20 mg/ml) was added 20 s after the addition of enzyme to give a final concentration of inhibitor between 0.05 and 0.3 μM. The absorbance at 340 nm was recorded for 10 min with each inhibitor at six different concentrations.

The rate constants of binding to and dissociation from F_1_-ATPase, *k*_on_ and *k*_off_, respectively, of each inhibitor protein were measured from the exponential decay of the rate of ATPase activity after the addition of various amounts of inhibitor protein. The change in absorbance of NADH with time is given by:

y(t)−y0=Vt∞+(((V0−V∞)/kinh)(1−exp(−kinht)))

where *y* is the absorbance at 340 nm, *t* is time, *k*_inh_ = *k*_on_[I] + *k*_off_, *V*_0_ is the initial rate of reaction, and *V*_∞_ is the rate of reaction at equilibrium.

In the presence of a large excess of inhibitor protein over F_1_-ATPase, the reaction rate constants become pseudo-first order, and thus the relationship becomes concentration dependent. Thus, *k*_inh_ varies with respect to [I], and the association and dissociation constants are determined from the straight line plot of *k*_inh_ = *k*_on_[I] + *k*_off_, where *k*_inh_ and *k*_off_, the rate constants for inhibitor dissociation, are expressed in inverse seconds; inhibitor concentration [I] is expressed as a molarity (μM); and *k*_on_, the rate constant for inhibitor binding, is expressed as μM^− 1^ s^− 1^.^8^ The dissociation constant for the inhibitor binding to enzyme, *K*_i_, was calculated from *K*_i_ = *k*_off_/*k*_on_, where *K*_i_ is the dissociation constant for inhibitor binding to enzyme, *k*_off_ is the rate constant for inhibitor dissociation, and *k*_on_ is the rate constant for inhibitor binding. The change in free energy of binding, ΔΔ*G*_binding_, brought about by the mutations, was calculated from ΔΔ*G*_binding_ = − *RT*ln(*K*_i_^wildtype^/*K*_i_^mutant^), where *K*_i_^wildtype^ and *K*_i_^mutant^ are the dissociation constants for the binding to the enzyme for the wild-type and mutated inhibitor proteins, respectively, and *R* is the gas constant and *T* the temperature in Kelvin. Sample analyses are given for the mutant inhibitor proteins F22Y I1-60GFP, E31A I1-60GFPHis, and F34A I1-60GFPHis in Supplementary Data. The decrease in ATPase activity as a result of an increase in inhibitor concentration is given (Supplementary Fig. S3) together with calculated apparent rate constants, *k*_inh_, for each inhibitor concentration (Supplementary Table S5) and plots of [I] *versus k*_inh_ (Supplementary Fig. S4).

### Structural data

Structural data concerning the bovine F_1_-I1-60 complex [PDB code 2v7q] are displayed in Figs. 1, 4, 5, and 7 with PYMOL.

## Footnotes

† http://www.embl.de/∼andrade/k2d.html
