# Supplementary material for: Binding of the Inhibitor Protein IF1 to Bovine F1-ATPase
Source: J Mol Biol. 2011 Feb 25;406(3):443–53. doi: 10.1016/j.jmb.2010.12.025 (PMC3041923; doi:10.1016/j.jmb.2010.12.025)
Supplement: Supplementary file 1 — Supplementary materials [file mmc1.pdf]

SUPPLEMENTARY DATA FOR:

Binding of the inhibitor protein IF_1 to bovine F_1-ATPase

John V. Bason, Michael J. Runswick, Ian M. Fearnley and John E. Walker

From The Medical Research Council Mitochondrial Biology Unit, Hills Road, Cambridge, CB0 2XY, United Kingdom

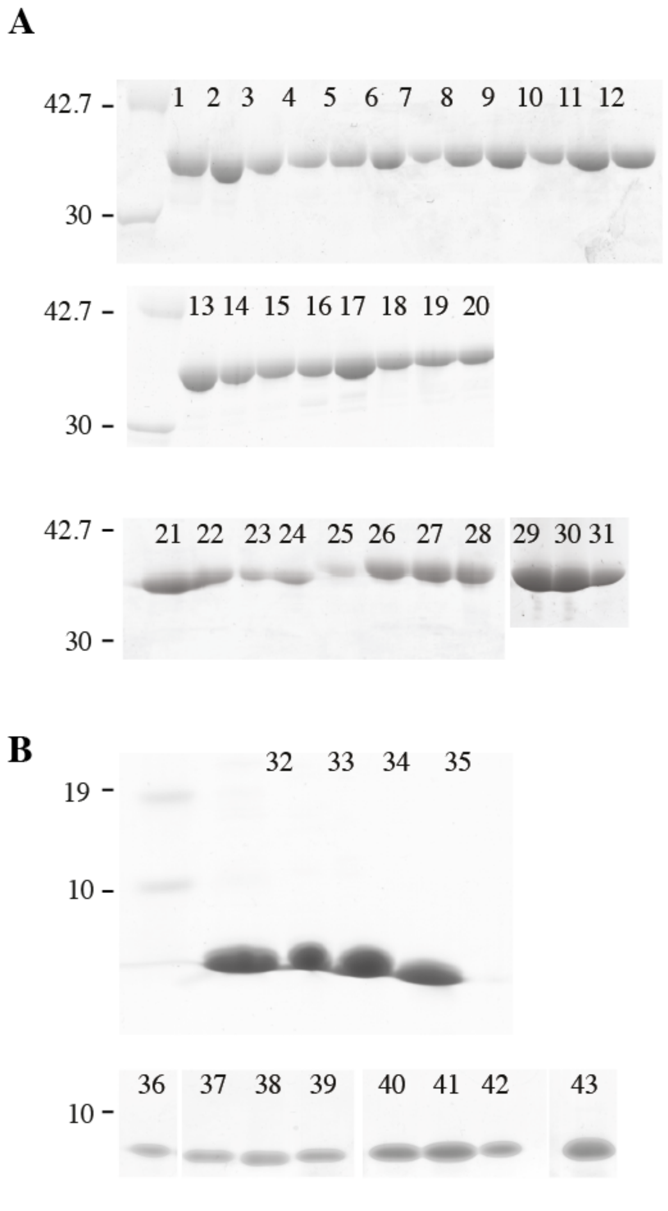

Supplementary Fig. 1. Characterization of bovine inhibitor proteins by SDS-PAGE. Part A. Lanes 1-35 contain residues 1-60 of bovine IF_1 (I1-60) fused via its C-terminus to GFP and hexa-histidine, with the following mutations in I1-60: 1, none; 2, F22A; 3, F22Y; 4, F22W; 5, K24A; 6, R25A; 7, E26A; 8, E29A; 9, E30A; 10, E31A; 11, R32A; 12, Y33A; 13, Y33W; 14, F34A; 15, F34Y; 16, R35A; 17, R37A; 18, Q41A; 19, L42A; 20, L45A; 21, Q27A; 22, K39A; 23, E40A; 24, Δ1-7; 25, Δ1-13; 26, Δ1-14; 27, Δ1-15; 28, Δ1-16; 29, A28V; 30, A43V; 31, A44V. Part B. Lanes 32-

43 contain I1-60 fused C-terminally directly to hexa-histidine with the following mutations; 32, A21G; 33, E30A; 34, Y33A; 35, R35A; 36, F22A; 47, R25A; 38, F34A; 39, Q41A; 40, L42A; 41, A43V; 42, A44V; 43, L45A. The positions of molecular weight markers are shown on the left.

Supplementary Table 1. Molecular masses of recombinant inhibitor proteins.

Masses were measured by ESI-MS, and calculated from protein sequences.

Protein	Mass (Da)		Mass difference	Modification
	Observed	Calculated		
I1-60GFPHis	34346.6	34344.8	+1.8	None
A21G I1-60GFPHis	34333.76	34330.73	+3.0	None
F22A I1-60GFPHis	34269.1	34268.7	+0.4	None
F22Y I1-60GFPHis	34361.4	34360.8	+0.6	None
F22W I1-60GFPHis	34348.9	34383.8	0.9	None
K24A I1-60GFPHis	34288.6	34287.7	+0.9	None
R25A I1-60GFPHis	34123.6	34259.7	-136.1	- His[a]
E26A I1-60GFPHis	34287.0	34286.7	+0.3	None
Q27A 1-60GFPHis	34290.8	34287.7	+3.1	None
A28V 1-60GFPHis	34374.5	34372.8	+2.3	None
E29A I1-60GFPHis	34286.8	34286.7	+0.1	None
E30A I1-60GFPHis	34289.0	34286.7	+2.3	None
E31A I1-60GFPHis	34288.0	34286.7	+1.3	None
R32A I1-60GFPHis	34260.1	34259.7	+0.4	None
Y33A I1-60GFPHis	34253.9	34252.7	+1.2	None
Y33W I1-60GFPHis	34369.5	34367.8	+1.7	None
F34A I1-60GFPHis	34269.3	34268.7	+0.6	None
F34Y I1-60GFPHis	34362.9	34360.8	+1.9	None
R35A I1-60GFPHis	34260.2	34259.7	+0.5	None
R37A I1-60GFPHis	34261.7	34259.7	+2.0	None
K39A 1-60GFPHis	34291.3	34287.7	+3.6	None

Supplementary Table 1 cont.

Protein	Mass (Da)		Mass difference	Modification
	Observed	Calculated		
E40A 1-60GFPHis	34290.3	34286.7	+3.6	None
Q41A I1-60GFPHis	34287.9	34287.7	+0.2	None
L42A I1-60GFPHis	34303.3	34302.7	+0.6	None
A43V 1-60GFPHis	34375.9	34372.8	+3.1	None
A44V 1-60GFPHis	34375.1	34372.8	+2.3	None
L45A I1-60GFPHis	34303.6	34302.7	+0.9	None
I1-60GFPHis Δ1-7	33699.4	33698.2	+1.2	None
	33829.9	33698.2	+131.7	+ M[b]
I1-60GFPHIS Δ1-13	33140.8	33140.2	+0.6	None
	33271.7	33140.2	+131.5	+ M[b]
I1-60GFPHIS Δ1-14	33070.2	33069.5	+0.7	None
	33200.5	33069.5	+131.0	+ M[b]
I1-60GFPHIS Δ1-15	33103.0	32970.4	+132.6	+ M[b]
I1-60GFPHIS Δ1-16	32945.9	32814.2	+131.7	+ M[b]
A21G I1-60His	7427.0	7427.1	-0.1	None
F22A I1-60His	7365.1	7364.9	+0.2	None
R25A I1-60His	7356.1	7355.9	+0.2	None
E30A I1-60His	7383.0	7382.7	+0.3	None
Y33A I1-60His	7348.9	7349.4	-0.5	None
F34A I1-60His	7364.9	7364.9	0.0	None
R35A I1-60His	7355.9	7356.1	-0.2	None

Supplementary Table 1 cont.

Protein	Mass (Da)		Mass difference	Modification
	Observed	Calculated		
Q41A I1-60His	7384.1	7384.0	+0.1	None
L42A I1-60His	7399.2	7399.0	+0.2	None
A43V 1-60His	7470.0	7469.1	+0.9	None
A44V 1-60His	7469.1	7469.1	0.0	None
L45A I1-60His	7399.8	7399.0	+0.8	None

[a] the loss of a histidine residue from the C-terminal histidine tag, did not affect purification of the protein by nickel affinity chromatography; [b,] the N-terminal methionine residue was retained.

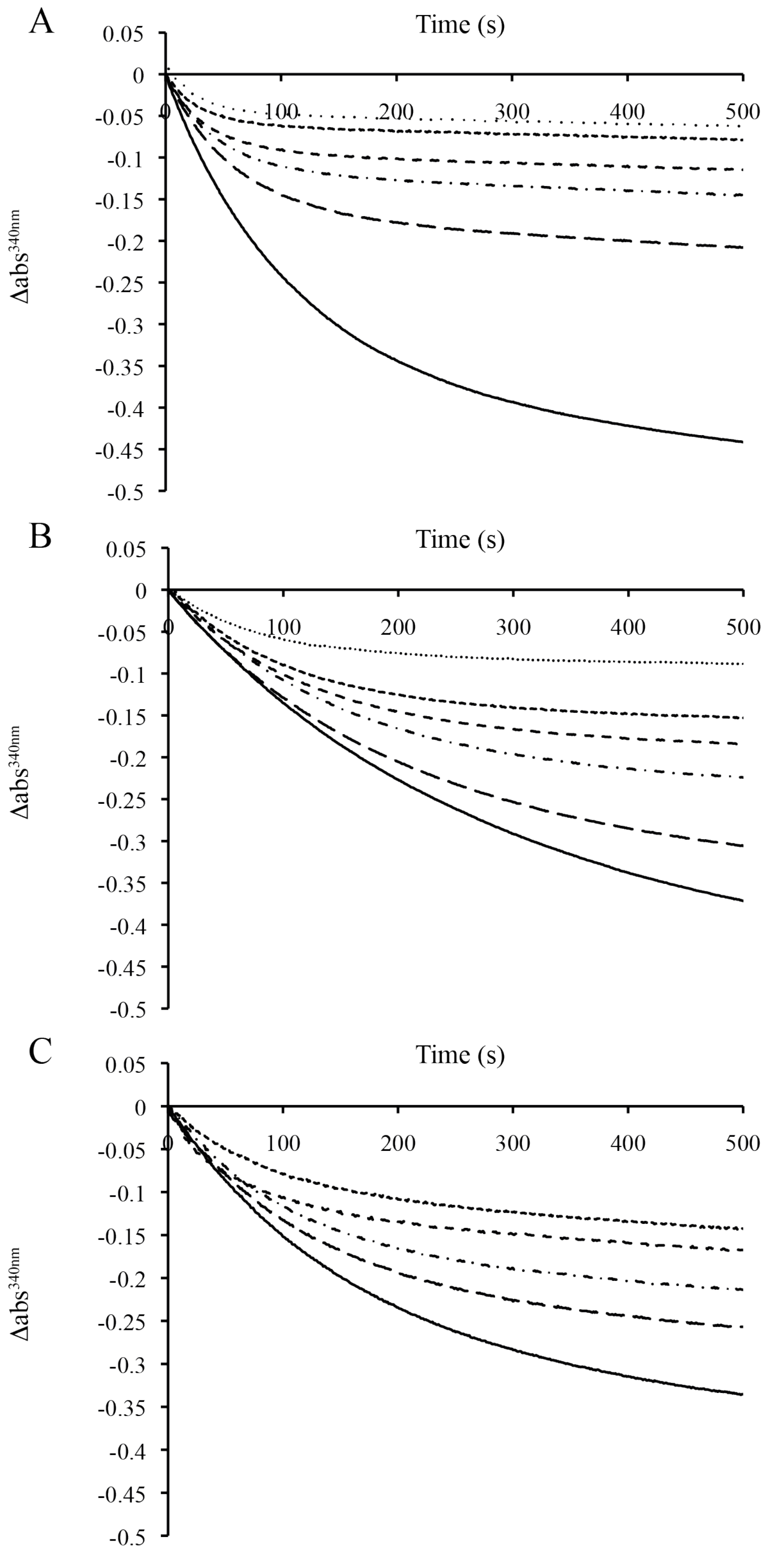

Supplementary Fig. 2. The decrease in ATPase activity of F_1-ATPase in response to increasing concentrations of the mutant inhibitor proteins. Part (A), F22Y I1-60GFPHis, part (B), E31A I1-60GFPHis; part (C), F34A I1-60GFPHis. The following concentrations of inhibitor were employed; in (A), 0.359 μM (….), 0.719

μM (----), 1.078 μM (- - -), 1.440 μM (__ __), 1.797 μM and 2.160 μM (___); in B. 0.1 μM (….), 0.15 μM (----), 0.2 μM (- - -), 0.25 μM (_._.), 0.3 μM (__ __) and 0.35 μM (___); in (C), 1.459 μM (----), 2.188 μM (- - -), 2.918 μM (for _._.), 3.648 μM (__ __) and 4.377 μM (___). The apparent rate constants, k_{inh} (see Supplementary Table 5) were calculated as described in Materials and Methods.

Supplementary Table 2. Calculated k_{inh} values for three mutant inhibitor proteins at various concentrations of the inhibitors (see Materials and Methods).

Protein	Concentration (μM)	k_{inh}
F22Y I1-60GFPHis	0.359	0.0091
	0.719	0.0168
	1.078	0.0231
	1.440	0.0277
	1.797	0.0336
	2.160	0.0385
E31A I1-60GFPHis	0.100	0.0041
	0.150	0.0058
	0.200	0.0066
	0.250	0.0087
	0.300	0.0102
	0.350	0.0117
F34A I1-60GFPHis	1.459	0.0066
	2.188	0.0082
	2.918	0.0100
	3.648	0.0119
	4.377	0.0140

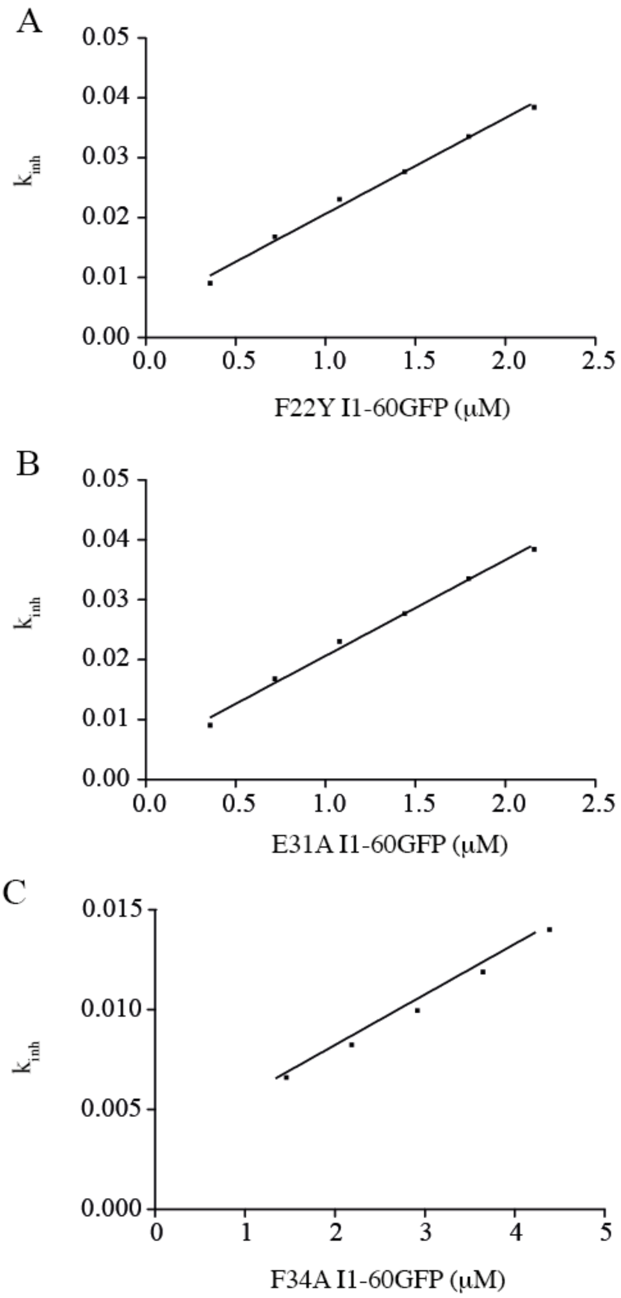

Supplementary Fig. 3. Dependence of the apparent rate constant, k_{inh} for the ATP hydrolase activity of F_1-ATPase at various concentrations of the mutant inhibitor proteins F22Y I1-60GFP (A), E31A I1-60GFPHis (B), and F34A I1-60GFPHis (C). The data are taken from Supplementary Table 2. As $k_{inh} = k_{on}[I] + k_{off}$, the values of k_{on} and k_{off} (see Supplementary Table 4) were obtained from the slopes of the graphs and the y intercepts, respectively.

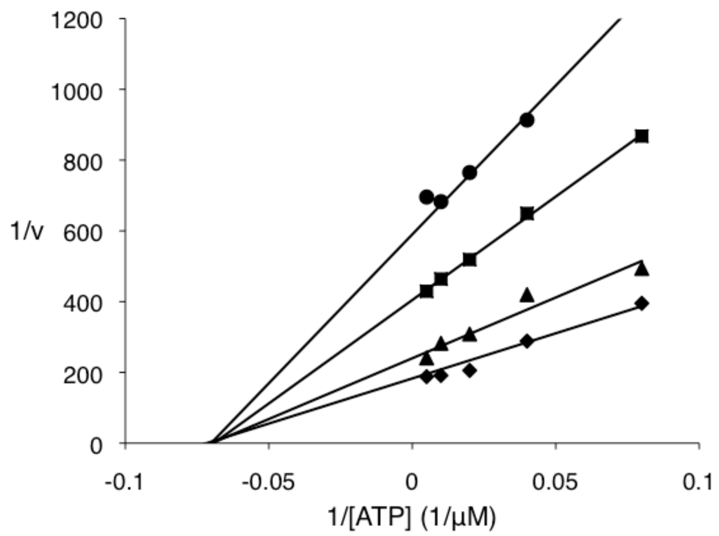

Supplementary Fig. 4. Analysis of the inhibition of F_1-ATPase by the inhibitor F22A I1-60GFPHis. The results are presented as a Lineweaver-Burke plot of 1/[ATP] versus 1/rate of reaction. The symbols ●, ■, ▲ and ◆ , correspond to measurements at 9.8, 7.5 μM, 4.9 and 2.45 μM ATP, respectively.

Supplementary Table 3. The binding and dissociation rate of reaction constants for N-terminally deleted inhibitor proteins.

Deletion	k_{on} ($\mu M^{-1}s^{-1}$) x 10^{-2}	k_{off} (s^{-1}) x 10^{-2}	K_i (μM^{-1}) x 10^{-2}	$K_i$wt: $K_i$mut	$\Delta\Delta G_{binding}$ (kcal mol^{-1})
None	2.60 ± 0.08	0.17 ± 0.01	6.50 ± 0.70	-	0.00
Δ1-7	5.30 ± 0.23	0.18 ± 0.03	3.50 ± 0.74	1.90	-0.38
Δ1-13	4.30 ± 0.31	0.36 ± 0.01	8.30 ± 0.84	0.78	0.15
Δ1-14	8.80 ± 0.83	1.70 ± 0.23	19.00 ± 4.40	0.34	0.67
Δ1-15	5.50 ± 0.89	2.00 ± 0.34	36.00 ± 12.00	0.18	1.05
Δ1-16	5.50 ± 0.91	2.00 ± 0.33	37.00 ± 12.00	0.17	1.08

Supplementary Table 4. The binding and dissociation rate constants for inhibitor proteins containing point mutations in the long α-helix of IF_1.

Mutation(s)	k_{on} x 10^{-2} ($\mu M^{-1}s^{-1}$)	k_{off} x 10^{-2} (s^{-1})	K_i x 10^{-2} (μM^{-1})	$K_i$wt: $K_i$mut	$\Delta\Delta G_{binding}$ (kcal mol^{-1})
None [a]	11.0 ± 0.47	0.32 ± 0.04	3.00 ± 0.57	-	-
None [b]	2.60 ± 0.08	0.17 ± 0.01	6.50 ± 0.70	-	0.000
A21G	0.55 ± 0.04	0.81 ± 0.07	150 ± 17.00	0.044	1.80
F22A[c]	nd	nd	250	0.026	2.30
F22Y	1.60 ± 0.07	0.46 ± 0.09	29.0 ± 7.10	0.230	0.92
F22W	2.20 ± 0.15	0.53 ± 0.10	24.0 ± 6.40	0.270	0.80
K24A	3.40 ± 0.09	0.20 ± 0.02	5.90 ± 0.81	1.100	-0.06
R25A	1.10 ± 0.05	1.50 ± 0.07	140 ± 13.0	4.700	1.90
E26A	4.40 ± 0.10	0.25 ± 0.02	5.80 ± 0.66	110.0	-0.075
Q27A	1.90 ± 0.12	0.46 ± 0.07	25.0 ± 5.70	0.260	0.83
A28V	1.20 ± 0.05	0.78 ± 0.06	63.0 ± 5.50	0.100	1.30
E29A	3.60 ± 0.06	0.33 ± 0.03	9.20 ± 0.92	0.710	0.21
E30A	no binding	no binding	-	0.000	-
E31A	2.80 ± 0.17	0.12 ± 0.03	4.20 ± 1.20	0.160	-0.27
R32A	3.20 ± 0.07	0.16 ± 0.02	4.90 ± 0.71	0.130	-0.18
Y33A	no binding	no binding	-	0.000	-
Y33W	10.00 ± 0.45	0.30 ± 0.06	2.80 ± 0.67	0.230	-0.51
F34A	0.25 ± 0.00	0.28 ± 0.02	110 ± 13.00	0.060	1.70

Supplementary Table 4 (cont).

Mutation(s)	k_{on} x 10^{-2} ($\mu M^{-1}s^{-1}$)	k_{off} x 10^{-2} (s^{-1})	K_i x 10^{-2} (μM^{-1})	$K_i$wt: $K_i$mut	$\Delta\Delta G_{binding}$ (kcal mol^{-1})
F34Y	1.00 ± 0.03	0.26 ± 0.04	25.0 ± 4.68	0.260	0.84
R35A	3.90 ± 0.08	0.13 ± 0.02	3.60 ± 0.62	0.180	-0.36
R37A	1.00 ± 0.04	0.16 ± 0.02	16.0 ± 2.26	0.410	0.55
K39A	1.20 ± 0.07	0.73 ± 0.04	62.0 ± 7.65	0.100	1.40
E40A	1.40 ± 0.10	0.82 ± 0.05	59.0 ± 8.10	0.110	1.36
Q41A	2.50 ± 0.13	0.49 ± 0.08	19.0 ± 4.20	0.330	0.68
L42A	0.39 ± 0.04	0.09 ± 0.005	23.0 ± 3.80	0.280	0.79
A43V	1.30 ± 0.09	0.76 ± 0.07	61.0 ± 7.11	0.110	1.30
A44V	0.10 ± 0.01	1.30 ± 0.10	1300 ± 16.32	0.050	3.10
L45A	0.18 ± 0.01	0.45 ±0.05	250 ± 44.33	0.260	2.20

[a] and [b], I1-60 without and with fused GFP and C-terminal hexahistidine tag, respectively; mutations were introduced into [b]; [c], because of the lack of exponential decay, k_{on} and k_{off} could not be determined, and so the K_i value was calculated from a Lineweaver-Burke plot; nd, not determined

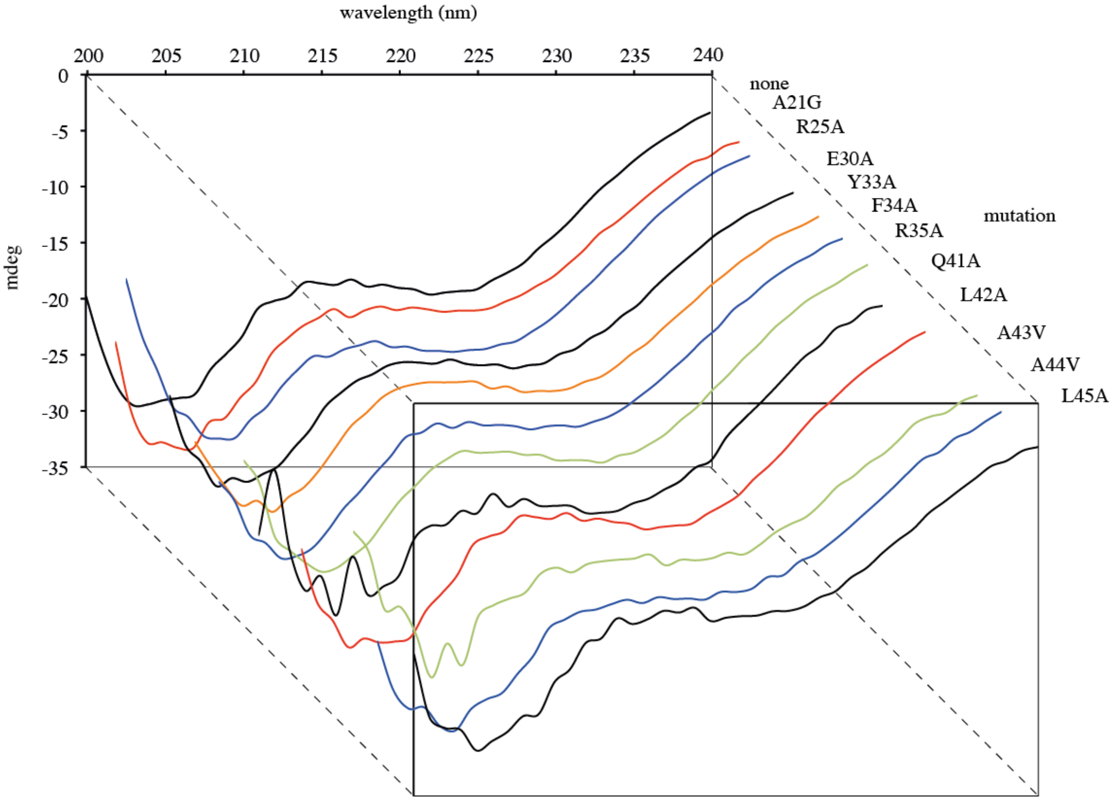

Supplementary Fig 5. Circular dichroism spectra of bovine I1-60His containing various point mutations in the longer α-helix. The proteins lack a GFP moiety.

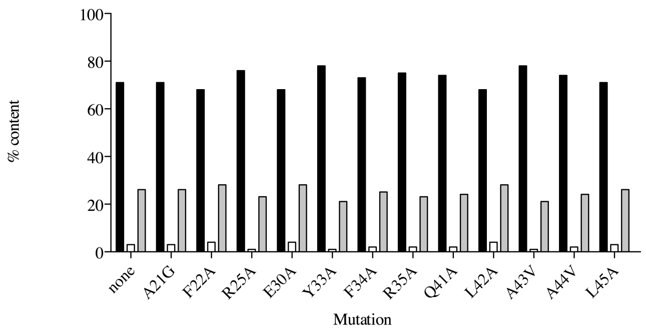

Supplementary Fig. 6. The secondary structure contents of mutant inhibitor proteins I1-60His. The percentage content of α-helix, β-sheet and random coil are given in black, white and grey, respectively.
